# Preparation and properties of Pue-loaded HA-ADH-PS nanomicelles

**DOI:** 10.1080/15685551.2020.1860481

**Published:** 2021-01-17

**Authors:** Huiru Wang, Yuanyuan Li, Yunpeng Min, Hang Zhang, Linkun Hao, Ru Zhang, Yunying Jiang, Yimin Song

**Affiliations:** a Department of Pharmaceutical Engineering, Qingdao University of Science and Technology, Qingdao P.R. China; b College of Marines Life Science, Ocean University of China, Qingdao, P.R., China

**Keywords:** puerarin, hyaluronic acid, nanomicelle, drug carrier

## Abstract

Puerarin (Pue) is the most abundant isoflavonoid in kudzu root. It has been widely used as a therapeutic agent for the treatment of cardiovascular diseases. However, poor-bioavailability of puerarin is the main obstacle to its widespread clinical applications. In this paper, HA-ADH-PS nanomicelles were prepared by chemical modification, noncovalent modification and etc, and characterized by means of FT-IR, ultraviolet (UV) and thermogravimetric analysis (TG). The encapsulation efficiency and drug loading of Pue-loaded HA-ADH-PS nanomicelles were 45.1% and 19.89% by UV, respectively. It could be observed from the transmission electron microscopy (TEM) images that HA-ADH-PS micelles appeared obvious spherical structure in the water. The particle size of HA-ADH-PS nanomicelles and Pue-loaded HA-ADH-PS nanomicelles were about 136.8 nm and 119.5 nm with a PDI of 0.237 and 0.272, respectively. The fluorescence probe method was used to characterize the critical micelle concentration, the critical micelle concentration (CMC) value of the nanomicells was 0.002 g/L and the results met the requirements and ensured the stability of micelles after dilution. DPPH assay suggested that Pue-loaded HA-ADH-PS nanomicelles had an obvious radical scavenging effect in vitro. MTT test showed that Pue-loaded HA-ADH-PS nanomicelles was non-toxic and had good biocompatibility. Thus, Pue-loaded HA-ADH-PS nanomicelles could be used as a potential drug carrier for puerarin.

## 1. Introduction

Puerarin(7, 4′-dihydroxyisoflavone-8β-glucopyranoside, Pue) as a natural isoflavone compound extracted from kudzu root, was demonstrated efficient effects in the expansion of coronary artery, reduction of vascular resistance and myocardial oxygen consumption, antithrombosis, elimination of free radicals and inhibition of the growth of cancer cells [1]. In addition, puerarin with low toxicity, wide range of security and good curative effects made it widely used in the treatment of cardiovascular and cerebrovascular diseases, including coronary heart disease, hypertension, angina, diabetes, sudden deafness and other diseases [2,3]. However, due to its poor water solubility(0.011 mol/L), liposolubility, absorption resulted from the puerarin which was similar in chemical constitution to soybean isoflavones, only 0.79% of the puerarin was expelled from the urine in the original form after 36 h of the oral administration of it to normal adults, and after 72 h，73.3% of it was excreted from faeces. As the result of that, its biological activity and efficacy were greatly restricted. Thus, its wide application in clinic was also limited [4,5]. Therefore, a lot of research work had been tried to improve the properties of puerarin at home and abroad, especially in recent years, there had been many reports on this field. Their researches had focussed on the bioavailability of puerarin or puerarin micronization or esterification of small molecular substances [6–8], but most of them were limited in vitro studies, the clinical data were insufficient. For this reason, what measures should be currently taken to improve the clinical efficacy of puerarin, reduce adverse reactions and expand its application scope were still one of the hot topics in the field of pharmacy.

In recent years, hyaluronic acid (HA), as an acidic mucopolysaccharide, had been widely used in the preparation of the novel drug delivery system. It had a targeted effects on tumour cells and could combine specifically with a variety of cell surface receptors, such as cluster of differentiation 44 (CD44), receptor of HA-mediated motility(RHAMM), lymphatic vessel endothelial HA receptor-1 (LYVE-1) and cell surface HA receptor (HARE), which were overexpressed on the surface of tumour cells [9–11]. In addition, HA presented good biocompatibility, low immunogenicity and biodegradability as the main component of the extracellular matrix and intracellular stroma [12,13]. Furthermore, HA could be obtained through different routes and it had a large number of functional groups such as carboxyl, hydroxyl and acetylamino groups, which could undergo structural modification by crosslink [14], esterification [15] and grafting [16]. HA with strong hydrophilicity was easy to dissolve in water but it was not easy to dissolve in organic solvents due to its strong intramolecular and intermolecular hydrogen bonding. The excellent properties of this natural polymer such as hydrophilicity, fluidity, viscoelasticity, targetability and biocompatibility had attracted the attention of pharmaceutical researchers all over the world.

In this paper, an ideal puerarin precursor with characteristics of amphiphilic Polymer Micelles（HA-ADH-PS polymer）, in which HA is as hydrophilic component and puerarin succinic anhydride (PS) is as hydrophobic component, adipic dihydrazide (ADH) is as linker, is prepared through chemical modification, noncovalent modification, and etc. Subsequently, under certain conditions, the precursor drug form hydrophobic polymer micelles with the unique structure, internal hydrophobicity and external hydrophilicity. And then the final target product, the Pue-loaded HA-ADH-PS nanomicelles is obtained by drug solubilization, ultrasonic dispersion, filtration and dialysis, freeze-drying, etc. What’s more, HA-ADH-PS nanomicelles are characterized by means of FT-IR, UV and TG and studied of the morphology. At last, encapsulation efficiency, drug loading, stability, in vitro release, DPPH-free radical action and biocompatibility are studied.

## 2. Materials and methods

### 2.1. Materials

Puerarin(Pue), made by our laboratory with a purity of 98%. Sodium hyaluronate (Mw = 10kDa), purchased from Shandong Zhengda Freida Co., Ltd. (Shandong, China); Adipic acid dihydrazide (ADH) was obtained from Aladdin ((Shanghai, China); Succinic anhydride was purchased from Tianjin Bodi Chemical Co., Ltd. (Tianjin, China); 1-(3-Dimethylaminopropyl)-3-ethylcarbodiimide hydrochloride (EDC), N-Hydroxysuccinimide (NHS) and 4-dimethylaminopyridine (DMAP) were obtained from Chengdou Kelong Chemical Reagent Factory(Chengdou, China); Mouse fibroblast cells were obtained from institute of oncology, Shanghai Jiaotong University; fetal bovine serum, specifications of analytical purity (AR), was obtained from Semerfer Biochemical Products (Beijing) Co., Ltd; DMEM, specifications of AR, by Gibco Company, USA; Other chemicals were analytical purity.

### 2.2. Preparation of pue-loaded nanomicelles

#### 2.2.1. Preparation of PS

**Figure 1.**
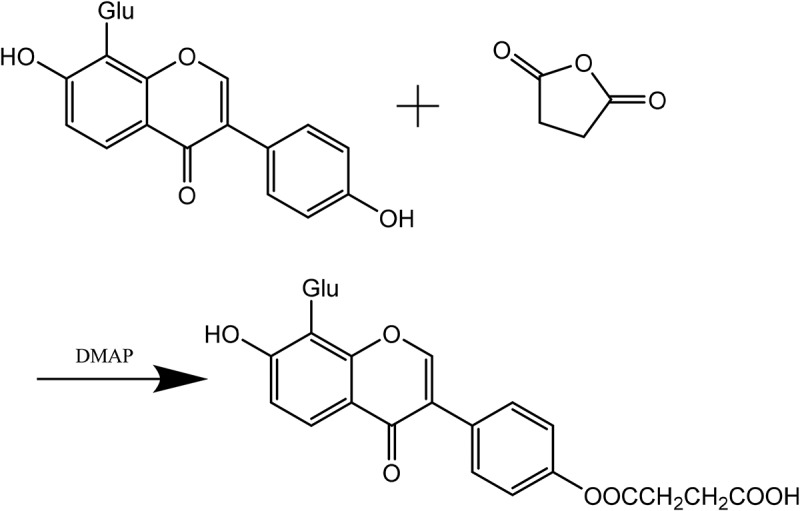
Esterification of puerarin with succinic anhydride

2.000 mg of puerarin were dissolved in 30 ml of anhydrous pyridine solution, followed by mixing well until it was dissolved completely. succinic anhydride and DMAP were added to reaction solution at 40°C for 5 h, followed by pouring a large amount of ether until crystallization. Filtering the reaction liquid and washing the cake with water. It was obtained the crude product of succinized puerarin after being vacuum-dried (LS-VO-50, shanghai, china). When the ratio of DMAP and succinic anhydride to puerarin was 0.33:1.5:1, the optimum reaction conditions were obtained, and the yield was 46.24%. Purification of the crude extract was by column chromatography. The crude product of succinized puerarin was discussed in methanol, the sample was added into a chromatographic column (4.5 cm × 80 cm), as eluent we used approximately a mixture of water: methanol: n-butanol (1:1:14), and collected required eluate. The eluate was added into the dialysis bags (Mwco 6000–8000) for 72 h. The solvent was evaporated using a rotary evaporator (RE-2000E, gongyi, China) The eluate was obtained by sehadex G 100 column chromatography. The puerarin succinate was obtained by dialysis for 72 hours and freeze-dried until constant weight. The reaction principle of Puerarin is shown in Figure 1.

#### 2.2.2. Preparation of HA-ADH

**Figure 2.**
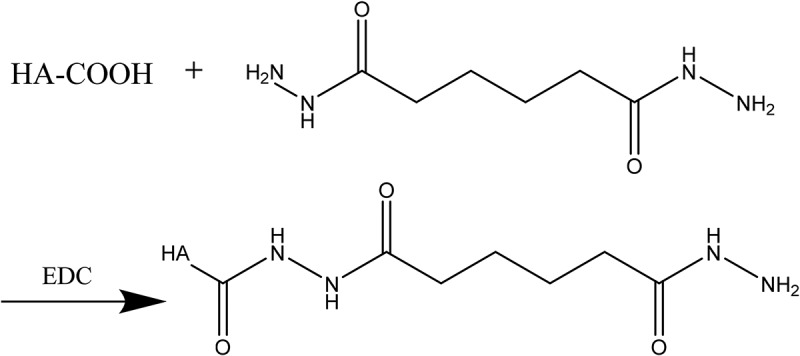
HA and cross-linking reaction of ADH

100 mg of Hyaluronic acid was dissolved in 25 mL of deionized water (pH 7) and magnetic stirred (ZZYYS, zhengzhou, china) for one day to activate full dissolution. HA solution with concentration of 4 mg/mL was prepared by adjusting pH between 4.0 and 6.0 with 0.1 M dilute hydrochloric acid. Afterwards, EDC (102 mg) and NHS (60 mg) were dissolved in 25 mL of water and stirred at room temperature for 1 h to activate the carboxyl, the hyaluronic acid activation solution was obtained. Appropriate amount of ADH was dissolved in deionized water and stirred to activate full dissolution. The above two solutions were mixed together and reacted for 2 h after the pH was adjusted to 7 with 0.1 M NaOH solution. When the ratio of hyaluronic acid: ADH: EDC was 1:10:2, the amount of amino and economic factors of the HA-ADH were the most suitable. Pure water was used for two days in a dialysis bag (Mwco 6000–8000). After being freeze-dried, HA-ADH was obtained. The cross-linking reaction of HA and ADH is shown in Figure 2.

#### 2.2.3. Preparation of HA-ADH-PS polymer

**Figure 3.**
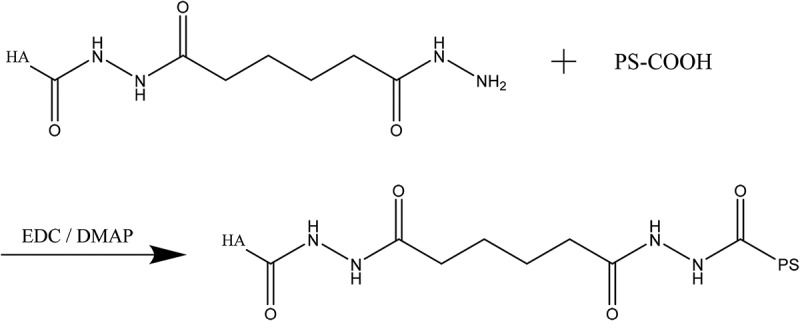
HA-ADH and cross-linking reaction of PS

20-mg HA-ADH and 80- mg PS were added into 40-ml deionized water and stirred to activate full dissolution. 40-mg EDC and 24-mg DMAP were added to react for 9 h at room temperature, appropriate deionized water was added to end the reaction. The sample was added into the dialysis bags (Mwco 6000–8000) for 2 days and changed water every 4–6 h. After being freeze-dried, HA-ADH-PS polymer was obtained. The cross-linking reaction of HA-ADH and PS is shown in Figure 3.

#### 2.2.4. Preparation of HA-ADH-PS nanomicelles

The form of HA-ADH-PS nanomicelles in aqueous solutions was triggered by the sonication of copolymer’s water solution by using a probe-type ultrasonic cell crusher (SJIA-150 W, Ningbo, china) in an ice bath. In detail, 15-mg HA-ADH-PS polymer was dissolved in 5 mL ultrapure water. The sample was stirred for 1 day and dialysed for 2 days. The dialysate was treated three times with probe-type ultrasonic cell grinder, 2.0 s of the working time, 4.0 s of the intermittent time, 20 times of the working times, and filtered through a 0.45 μm membrane filter.

#### 2.2.5. Preparation of pue-loaded HA-ADH-PS nanomicelles

15-mg HA-ADH-PS was dissolved in 5 mL ultrapure water and 10-mg puerarin was dissolved in 5 mL ethanol. Then, puerarin was slowly dripped into HA-ADH-PS suspension. The sample was stirred for 1 day and dialyzed for 2 days. Dialysate was treated three times with probe-type ultrasonic cell grinder, 2.0 s of the working time, 4.0 seconds of the intermittent time, 20 times of the working times, and filtered through a 0.45 μm membrane filter.

### 2.3. Structural characterization of puerarin derivatives

#### 2.3.1 Melting point

The melting points of puerarin and puerarin derivatives were detected by micro melting point apparatus (XT4A, Beijing, China). The sample was dried to constant weight and ground into powder. Then, clamping a small amount of sample with two cover slides and placing it on the heating table. Next, turn on the lamp and directly illuminate the heating table. At the beginning of heating, the temperature was increased at 20°C/min until 160°C, then the heating rate was decreased and 2°C/min. The initial melting temperature and full melting temperature were recorded.

#### 2.3.2 Ultraviolet (UV)

Taking water as blank solution, the absorbance of Puerarin solution and puerarin derivative solution in the wavelength range of 190–800 nm was scanned to obtain the UV Vis absorption spectrum.

#### 2.3.3 Fourier transform infrared spectrometer (FT-IR)

The appropriate amount of Pue, PS, HA, HA-ADH, HA-ADH-PS polymer were, respectively, mixed with 100 mg KBr, and the thin films were prepared by pressing method. Then the samples were analyzed by Bruker tensor-27 Fourier Transform Infrared Spectrometer (TENSOR, Germany) at wavelengths in the range of 400–4000 cm^−1^

### 2.4. Thermogravimetric analysis (TGA)

The thermal stability was determined by thermogravimetric analysis (TGA) (TGA, Mettler-Toledo). Thermogravimetric curves (TG) of HA, ADH, SA, Pue and HA-ADH-PS polymers were measured by thermogravimetric analysis while the apparatus was continually flushed with anitrogen flow. Samples of 5.0 (±0.1) mg were spread evenly in an alumina crucible for each analysis and heated at rate of 10 °C/min, respectively, from 25 °C to 700 °C.

### 2.5. Micelles size, zeta potential and PDI

The particle size, zeta potential and PDI of HA-ADH-PS nanomicelles and Pue-loaded HA-ADH-PS nanomicelles at the concentration of 1 mg/mL were evaluated with a Laser particle size analyzer (MD-1, Chongqing, china).The determination conditions were set as detection angle of 90°, temperature of 25°C, and λ = 532 nm.

### 2.6. Morphology measurement

The morphology of nanomicelles was investigated using a transmission electron microscopy (TEM, JEM-2100, JOEL, Tokyo, Japan). The samples were deposited on a carbon film-coated copper grid and stained with phosphotungstic acid aqueous solution (2%, w/v), then dried at room temperature and observed HA-ADH-PS nanomicelles and Pue-loaded HA-ADH-PS nanomicelles under TEM.

### 2.7. Standard curve of puerarin

Accurate 10-mg standard sample of puerarin was first put into a 100 mL volumetric flask; then the buffer of pH 6.8 and 7.4 and the artificial gastric juice of pH 1.2 were added to the scale, respectively, followed by shaking the mixture until it was homogeneous. Aliquots of solutions (0.2, 0.4, 0.6, 0.8 and 1.0 ml) were taken from the flask and placed into 10 ml volumetric flasks. Then, the buffer of pH 6.8 and 7.4 and the artificial gastric juice of pH 1.2 were added to the scale, respectively. The samples were measured by UV (752 N, shanghai, china) at 250 nm wavelength.

**Figure 4.**
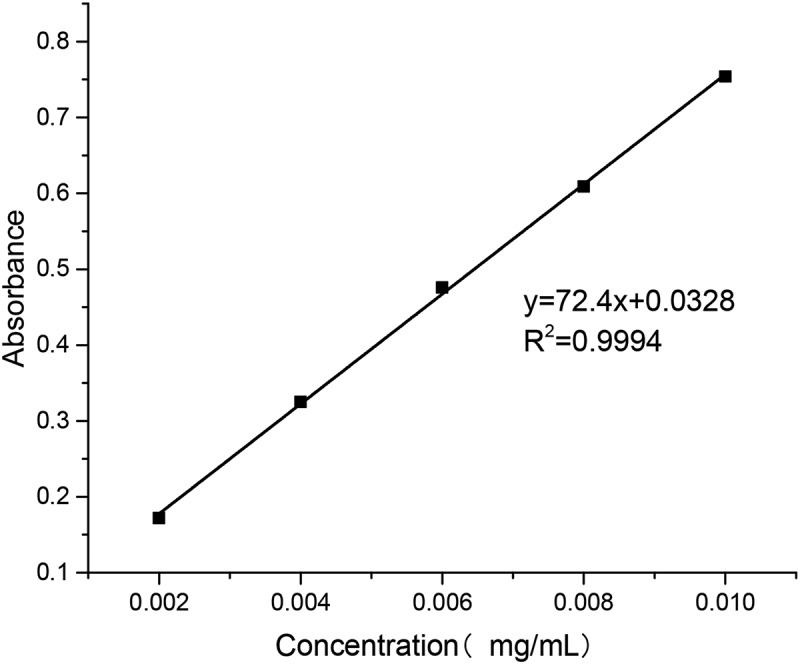
Puerarin standard curve using pH 6.8 PBS buffer as solvent

As shown in Figure 4, the relationship between puerarin concentration and absorbance in pH 6.8 PBS buffer was obtained by data processing.

$$y=72.4x+0.0328,\;R^{2}=0.9994$$

**Figure 5.**
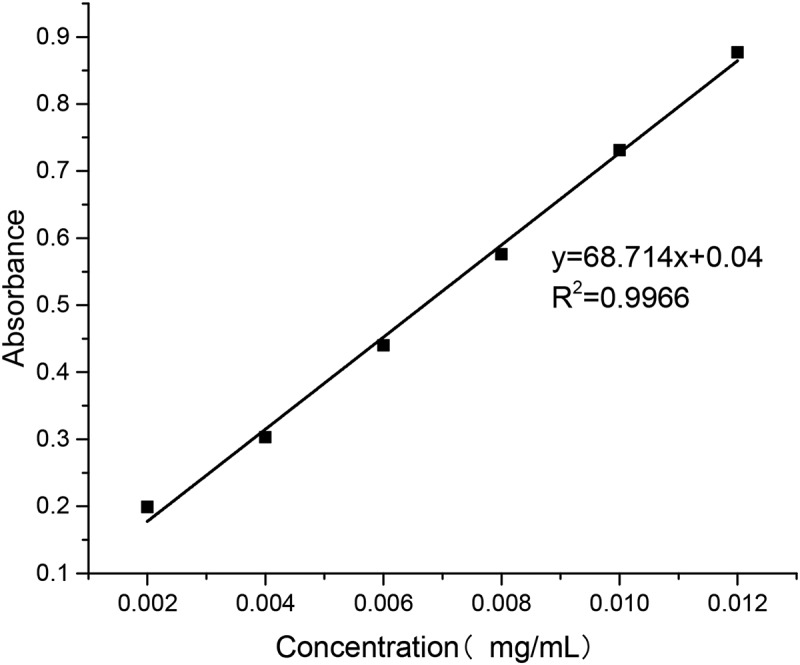
Puerarin standard curve using pH 7.4 PBS buffer as solvent

As shown in Figure 5,   the relationship between puerarin concentration and absorbance in pH 7.4 PBS buffer was obtained by data processing.

$$y=68.714x+0.04,\;R^{2}=0.9966$$

**Figure 6.**
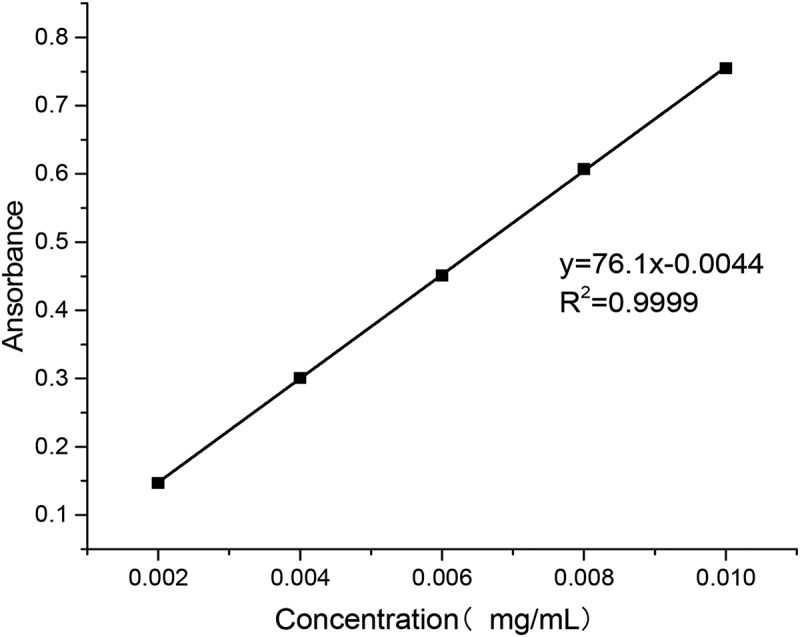
Puerarin standard curve using artificial gastric juice as solvent

As shown in Figure 6, the relationship between puerarin concentration and absorbance in artificial gastric juice was obtained by data processing.

$$y=76.1x-0.0044,\;R^{2}=0.9999$$

### 2.8. Entrapment efficiency (EE) and drug loading (DL)

After dialysis, Pue-loaded HA-ADH-PS nanomicelles were further broken up completely by centrifuging at 10,000 r/min for 15 min and obtained the supernatant. Recording the absorbancy of the supernatant at 250 nm, the amount of puerarin in the supernatant was analyzed by drawing standard curve above. DL and EE were calculated by following equations:

$$EE\left(\%\right)=\frac{Weight\;of\;drug\;in\;nanomicelles}{Weight\;of\;added\;drug}\times 100$$

$$DL\left(\%\right)=\frac{Weight\;of\;drug\;in\;nanomicelles}{Weight\;of\;nanomicelles}\times 100$$

### 2.9. Critical micelle concentration (CMC) measurement

The critical micelle concentration (CMC) of nanomicelles was determined using a fluorescence spectrophotometer (F-7000, Hitachi, Japan) with pyrene (1,6-diphenyl-1,3,5-hexatriene, DPH) as probe [17]. The determined concentration of nanomicelles was ranged from 9.7 × 10 ^−4^ to 0.5 mg/mL and the pyrene concentration was diluted to 2 × 10^−6^ M. The samples were set for a day at room temperature. The emission spectra of the nanomicelles were recorded from 350 to 450 nm with an excitation wavelength of 394 nm. The excitation and emission slit widths were 5 nm and 2.5 nm, respectively. Scanning at 500 m/min. Peak height intensity ratio (I1/I3) of the first peak (I1 at 375 nm) to the third peak (I3 at 386 nm) was plotted against the logarithm of nanomicelles concentration. The CMC value was taken from the intersection of the tangent to the curve at the inflection with the horizontal tangent through the points at low concentrations.

### 2.10. Nanomicelles stability studies

The stability of nanomicelles was investigated by the accelerated stability test. The nine dried samples of Pue-loaded HA-ADH-PS nanomicelles were placed in a constant temperature and humidity chamber at 40°C (± 2) and 75 % (± 5) relative humidity for 3 months. The stability of the micelles was monitored by the cumulative release in the samples within 72 hours. Moreover, the release properties of samples were tested at the end of the 1st, 2nd and 3rd month at pH 6.8 PBS. The same sample was operated three times in parallel.

### 2.11. In vitro release

The vitro release study of puerarin from Pue-loaded HA-ADH-PS nanomicelles was performed via dialysis technology. 5-mg Pue-loaded HA-ADH-PS nanomicelles were dissolved in buffers of 5 mL pH 6.8 and 7.4 and artificial gastric juice of 5 mL pH 1.2, respectively. The samples were added into the dialysis bags and submerged in 10 mL of solvents. The samples were placed in the 50 mL Ep tubes and the Ep tubes were placed on the shaker to oscillate at an appropriate speed at 37°C, in addition, the buffer of pH 6.8, pH 7.4 and the artificial gastric juice of pH 1.2 were replaced at 0.25, 0.5, 1, 2, 4, 6, 8, 12, 24, 48 h. Ultraviolet spectrophotometer was used to measure the absorbance, and the formula was obtained by drawing standard curve. The quality of puerarin released from Pue-loaded HA-ADH-PS nanomicelles in each time period was calculated, the release curve of puerarin was made, and the release type was judged.

### 2.12. The radical scavenging activity of pue-loaded HA-ADH-PS nanomicelles

The radical scavenging activity of Pue-loaded HA-ADH-PS nanomicelles was determined by 2, 2-diphenyl-1-picrylhydrazine (DPPH) assay. A determined concentration sample solution was mixed with 4 mL 0.1 mM/L DPPH and methanol solution and left at room temperature in the dark for 30 minutes. Then, the absorbance of the resulting solution was measured at 517 nm with UV–vis assay. Moreover, used as the negative control was vitamin C and the blank was distilled water. The DPPH radical scavenging activity was calculated by the following equation:

$$DPPH\;radical\;scavenging\;activity\;\left(\%\right)=\frac{A_{0}-A_{1}}{A_{0}}\times 100\%$$

Where A_0_ is the absorbance of the blank, A_1_ is the absorbance of the sample.

### 2.13. Biocompatibility assay

3-(4, 5-dimethylthiazol-2-yl)-2,5-diphenyltetrazolium bromide (MTT)was used to evaluate the cytotoxicity of biomaterials in vitro [18]. L-929 cells (mouse fibroblasts) in logarithmic growth phase were selected for MTT test. The cells were digested to prepare cell suspension. In Dulbecco’s Modified Eagle’s Medium (DMEM) containing 10% fetal bovine serum. Mouse fibroblasts in logarithmic growth phase were harvested and diluted to a concentration of 1 × 10^4^ cell/mL with the medium. Added in each well in 96-well plates was 100 μl cellular solution, and incubation was performed in an incubator (NU-4850, NuAire, Plymouth, MN, USA) at 37°C for 24 h under humidified atmosphere containing 5% CO2. The medium was then replaced with 100 μl sample solution. After 24 h, 48 h, and 72 h culture, the sample solution was replaced with 80 μl DMEM containing 8% fetal bovine serum, and 20 μl MTT at 5 mg/mL was added. The whole medium was removed after 4-h incubation, and 150-μl dimethyl sulfoxide (DMSO) was added. After 10-min shaking, the optical density (OD) was measured at 490 nm using microplate reader (Elx800, BioTek, Winoo-ski, VT, USA). Used as the negative control was 100 μl medium, and 100 μl of 5% phenol solution was used as the positive control.

The relative growth ratio (RGR) was determined as follows:

$$RGR\left(\%\right)=\frac{OD_{test}-OD_{negative}}{OD_{positive}-OD_{negative}}\times 100$$

The cytotoxicity is noted in six different levels according to the RGR value, as shown in Table 1.

**Table 1.** Relationship between the RGR value and cytotoxicity level

RGR(%)	Leavel
>100	0
75–99	1
50–74	2
25–49	3
1–24	4
0	5

### 2.14. Statistical analysis

Statistical analysis was conducted with the Origin 8 software, and evaluation was carried out using the one-way analysis of variance. All data were expressed with mean values and corresponding standard deviation values (mean +SD). P < 0.05 indicates statistical significance.

## 3. Results and discussion

### 3.1. Structural characterization of puerarin derivatives

#### 3.1.1. Melting point

As shown in Table 2, the melting point of puerarin derivative was significantly lower than that of puerarin. The melting point of puerarin derivatives decreased, indicating that the product was not puerarin itself. The melting point of succinic anhydride is 119.6 °C. The decrease of melting point of puerarin derivative was presumed to be the reaction of phenolic hydroxyl group with succinic anhydride, and intermolecular hydrogen bond decreases.

**Table 2.** Melting point determination results of puerarin and puerarin derivatives

Sample	Initial melting temperature	Full melting temperature	Melting range
Puerarin	205	211	6
puerarin derivatives	188	193	5

##### 3.1.2. UV

**Figure 7.**
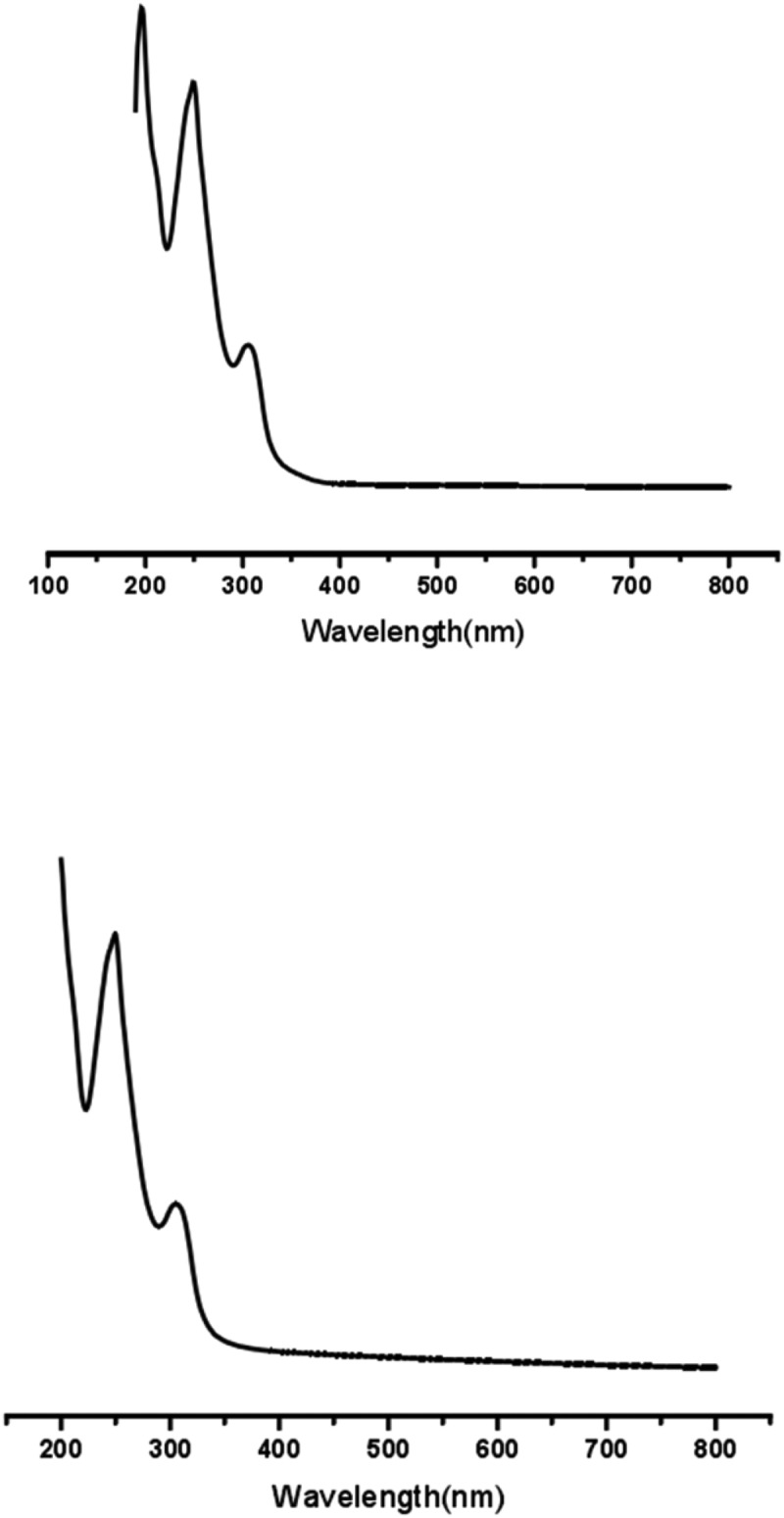
UV-visible absorption spectra: (a) puerarin. (b) puerarin derivatives

As showed in Figure 7, the UV spectra of puerarin and puerarin derivatives showed similar characteristic absorption, indicating that the basic structure of puerarin derivatives did not change, and the isoflavone ring was complete. The absorption peaks of them were 196.0 nm, 249.0 nm and 306.0 nm, respectively. Among them, the absorption peak of 249.0 nm was due to the conjugation of two 兀bonds in -C = C-C = O- structure, which was the characteristic absorption of B ring of puerarin. The absorption peak of 196.0 nm was the role of double bonds in benzene ring. And the absorption peak of 306.0 nm was caused by the conjugation of three double bonds in benzene ring, which overlapped with the vibration of benzene ring.

##### 3.1.3. FT-IR

**Figure 8.**
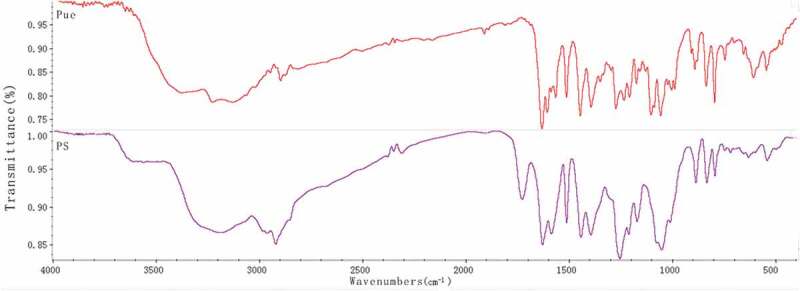
FT-IR of puerarin and puerarin derivatives

Figure 8 shows the FT-IR spectra of puerarin, and puerarin derivatives, respectively. As depicted in Figure 8, the FT-IR spectrum of puerarin, stretching vibration at 3376.89 cm^−1^ was the O-H group from associated alcohols and phenols; the anti-symmetric stretching vibrations at 2950.03 cm^−1^ and 2900.06 cm^−1^ were the C-H; stretching vibrations at 1634.19 cm^−1^ and 1514.97 cm^−1^ were the C = C group from A ring and C ring of benzene; the bending vibration at 1448.06 cm^−1^ was the 6, CH_2_ of the glucopyranose ring; stretching vibration from 1275.06 cm^−1^ to 1005.48 cm^−1^ were ascribed to the O-H of B ring and glucopyranose ring; stretching vibration at 1058.71 cm^−1^ was the 6, secondary alcohol of glucopyranose; stretching vibration at 893.44 cm^−1^ was the O-H group. Compared with the infrared absorption of puerarin, the infrared spectra of succinylated puerarin changed obviously. The broad peak at 3193.17 cm^−1^ was weakened and the hydroxyl group in the product was reduced. A new peak appeared at 1728.88 cm^−1^, it was succinified puerarin linked to carboxyl group, and a new peak at 1256.16 cm^−1^ was presumed to be esterification reaction to form ester bond. These results indicated that the hydroxyl group of puerarin reacted with succinic anhydride, with a new carboxyl group.

**Figure 9.**
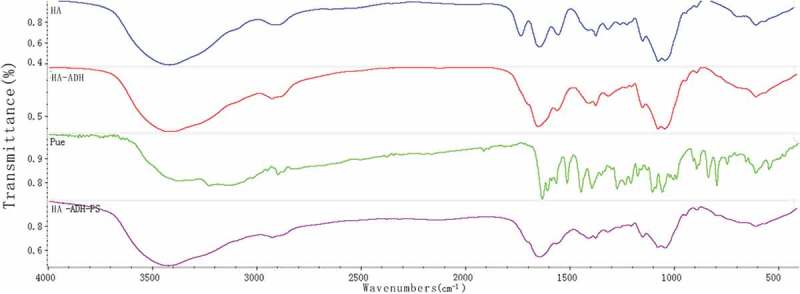
FT-IR of HA, Pue, HA-ADH and HA-ADH-PS

As shown in Figure 9, compared with the infrared spectra of hyaluronic acid, C = O stretching vibration of carboxylic acid at 1737.17 cm^−1^ disappeared, while C = O stretching vibration and N-H bending vibration at 1645.82 cm^−1^ increased. The results showed that the carboxyl group of hyaluronic acid participated in the amide reaction and formed amide bonds. At the same time, puerarin derivatives also linked hyaluronic acid with amide bonds. In addition, the symmetrical and antisymmetrical stretching vibrations at 1152.17 cm^−1^ and 1208.08 cm^−1^ were attributed to C-O-C of ester, which indicated that puerarin phenolic hydroxyl group had reacted with succinic anhydride and was linked by ester bond. The characteristic absorption peak of puerarin appeared at 795.47 cm^−1^. By combining the above FT-IR spectrum analyses, it was proved that hyaluronic acid and puerarin were successfully linked by ester bond and amide bond.

### 3.2. Thermogravimetric analysis

**Figure 10.**
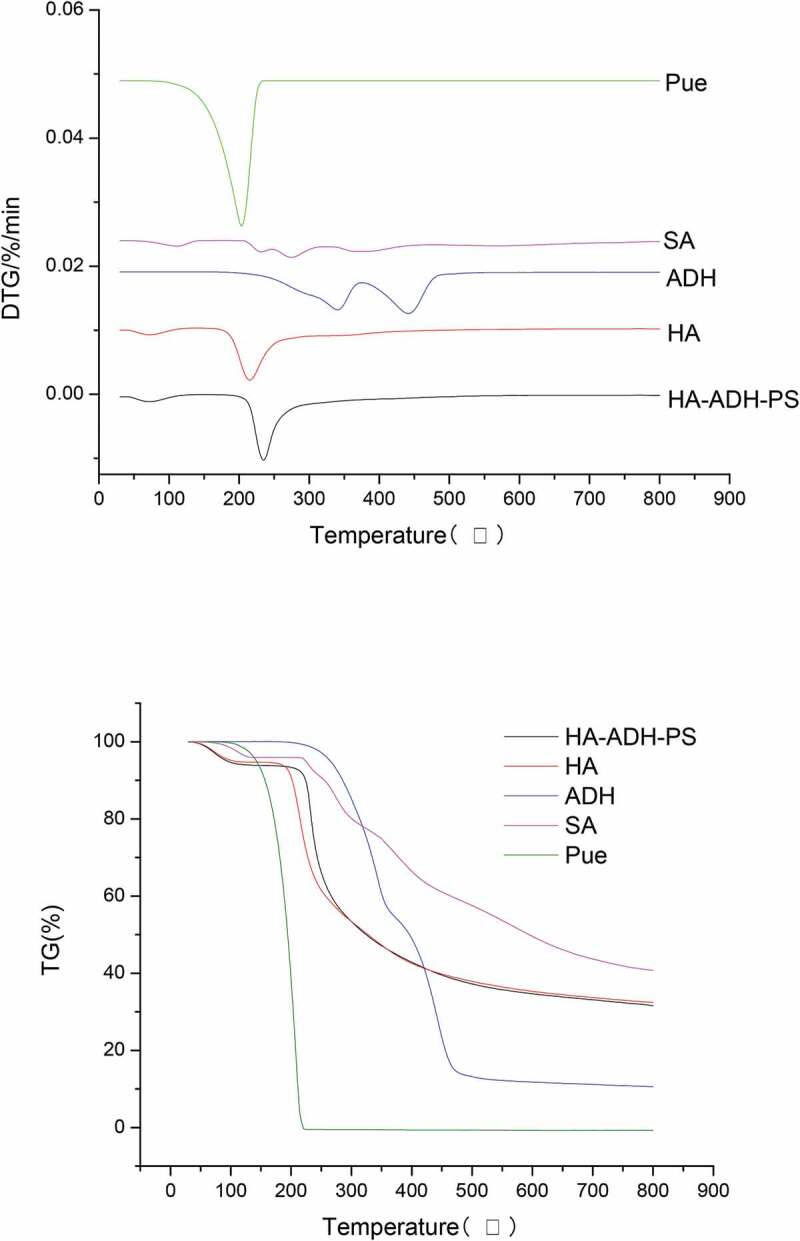
Thermogravimetric analysis (TG) and differential thermogravimetric analysis (DTG) curves for raw materials and HA-ADH-PS

TG-DTG curves of the samples are shown in Figure 10. It could be seen from TG-DTG curves that the curves of the HA had two stages. The first region was from 42.23°C to 130.95°C with a weight loss of 5.1553% and a maximum decomposition temperature of 74.51°C, which was mainly caused by the loss of water attached to hyaluronic acid and other volatile compounds. The second stage (162.60–554.41°C) was the main period with a weight loss of 62.2674% and the maximum decomposition temperature of 221.01°C.

It could be seen from TG-DTG curves that the curves of the HA-ADH-PS also had two stages. The first stage was from 41.51°C to 137.41°C with a weight loss of 6.1324% and a maximum decomposition temperature of 74.56°C, which was mainly caused by the decomposition of crystalline water, volatile compounds and connected puerarin. The second stage (186.86–537.76°C) was the main period with a weight loss of 62.1146% and the maximum decomposition temperature of 241.81°C. The analysis showed that the maximum decomposition temperature of HA-ADH-PS was higher than HA, the maximum decomposition temperatures of adipic dihydrazide in the two-step decomposition were 356.34°C and 458.80°C, respectively, the maximum decomposition temperature of succinic anhydride was 211.39°C, the maximum decomposition temperature of puerarin in the main decomposition stage was 284.28°C, so the thermal stability of HA-ADH-PS was enhanced when the three raw materials were connected to hyaluronic acid.

### 3.3. Particle size, PDI and morphology

**Figure 11.**
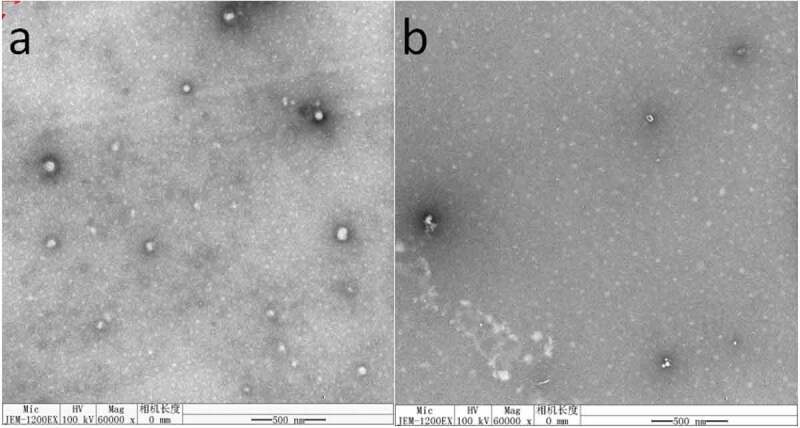
TEM images: (**a)** HA-ADH-PS nanomicelles; (**b)** Pue-loaded HA-ADH-PS nanomicelles

**Figure 12.**
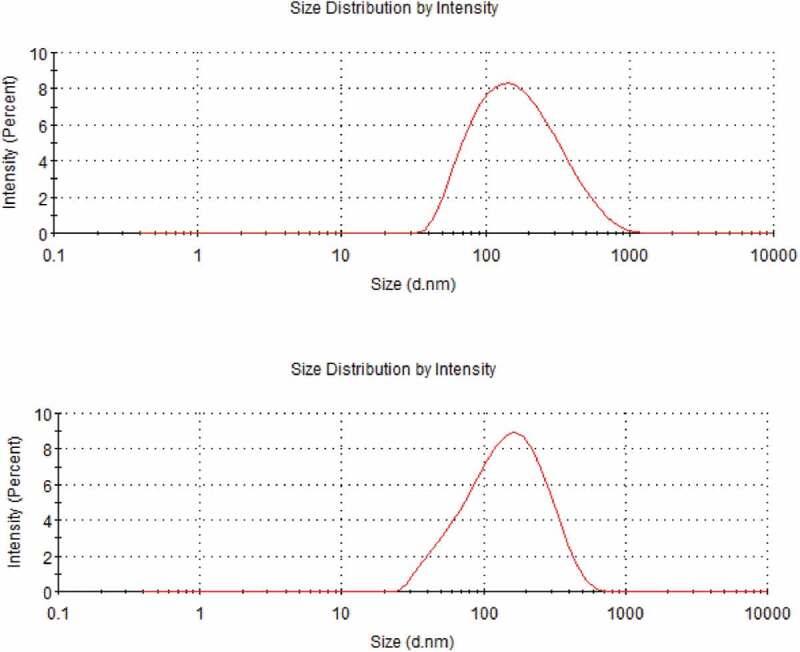
Particle size results: (a) HA-ADH-PS nanomicelles; (b) Pue-loaded HA-ADH-PS nanomicelles

Figure 11 shows the morphology of nanomicelles. In water medium (pH = 7), the hydrophilic chain was exposed and came into contacting with water, while the hydrophobic ends got together around the drug to form a spherical structure. The diameter of HA-ADH-PS nanomicelles was about 65 nm and the diameter of Pue-loaded HA-ADH-PS nanomicelles decreased to about 57 nm. It is because that the hydrophobicity of the core was enhanced and the micelles were filled more tightly after puerarin filled the hydrophobic core, thus reducing its size. Particle size of nanomicelles was illustrated in Figure 12. The particle size of HA-ADH-PS nanomicelles and Pue-loaded HA-ADH-PS nanomicelles were about 136.8 nm and 119.5 nm with a PDI of 0.237 and 0.272, respectively.

### 3.4. Encapsulation efficiency (EE) and drug loading (DL)

The encapsulation efficiency (EE) and drug loading (DL) are two important parameters that influences the drug-loaded nanomicelles. The EE and DL of Pue-loaded HA-ADH-PS nanomicelles were indirectly obtained by the measurement of the samples absorption using a UV spectrophotometer at 250 nm. As shown in Table 3, the EE% and DL% for Pue-loaded HA-ADH-PS nanomicelles were around 45.1% and 19.89, respectively.

**Table 3.** Encapsulation efficiency and drug loading of Pue-loaded HA-ADH-PS nanomicelles

Sample	Sample 1	Sample 2	Sample 3	Average
EE	44.97	45.04	45.29	45.1
DL	19.79	19.85	20.03	19.89

### 3.5. Critical micelle concentration (CMC)

**Figure 13.**
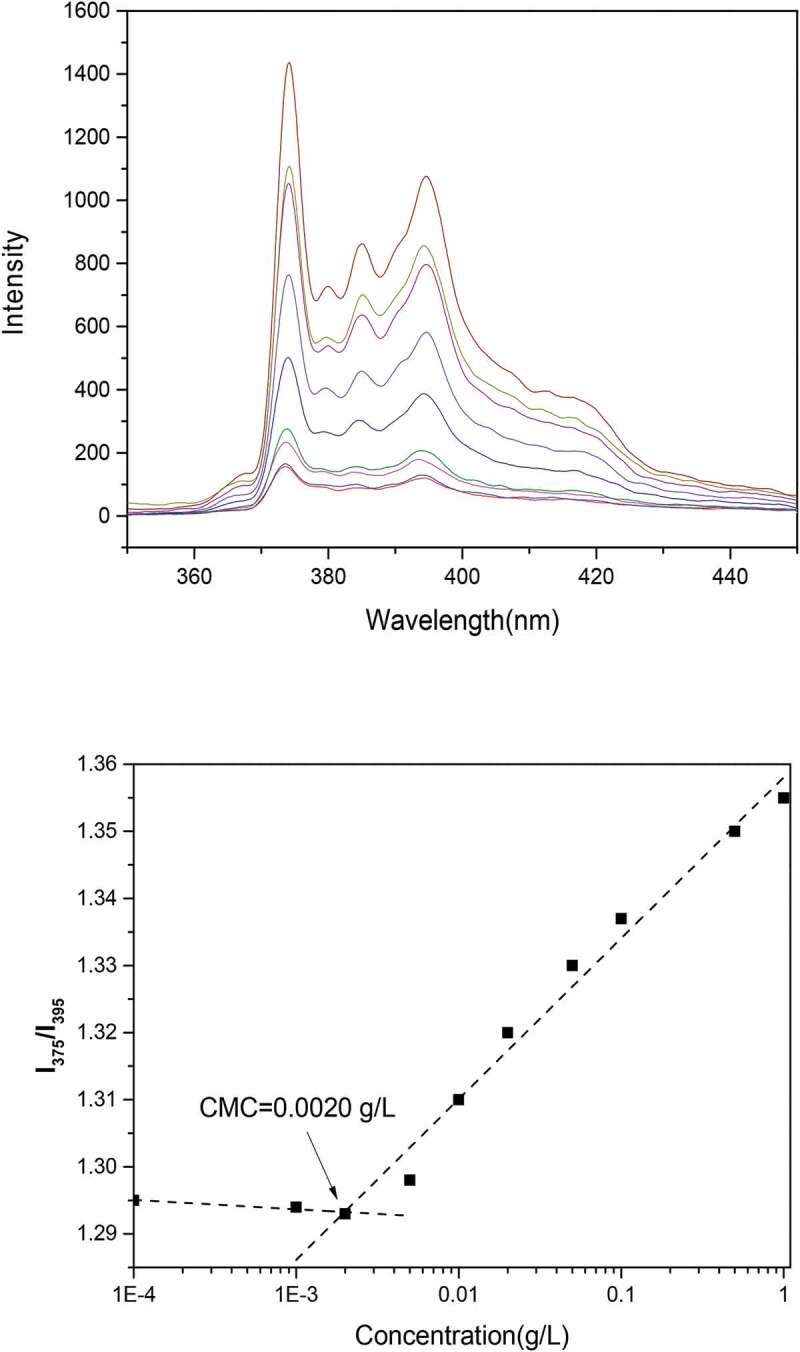
(a) Fluorescence emission spectra with increasing Pue-loaded HA-ADH-PS nanomicelles concentration; (b) Intensity ratio (I375/I395) plotted against Pue-loaded HA-ADH-PS nanomicelles concentration

The critical micelle concentration (CMC) is an important parameter that influences the micellar stability. Only with low CMC value can the micellar stability after administration in the body and dilution in bloodstream be guaranteed [19]. Figure 13（a） shows the emission spectra of pyrene at different concentrations. The increase of fluorescence intensity and red shift was observed with the increase of pyrene concentration. Figure 13 (b) shows the I375/I395 vs. log C plots of Pue-loaded HA-ADH-PS nanomicelles. A CMC value of 0.002 g/L was obtained from the cross-over point of the plots. The results met the requirements and ensured the stability of micelles after dilution.

### 3.6. Stability study

**Figure 14.**
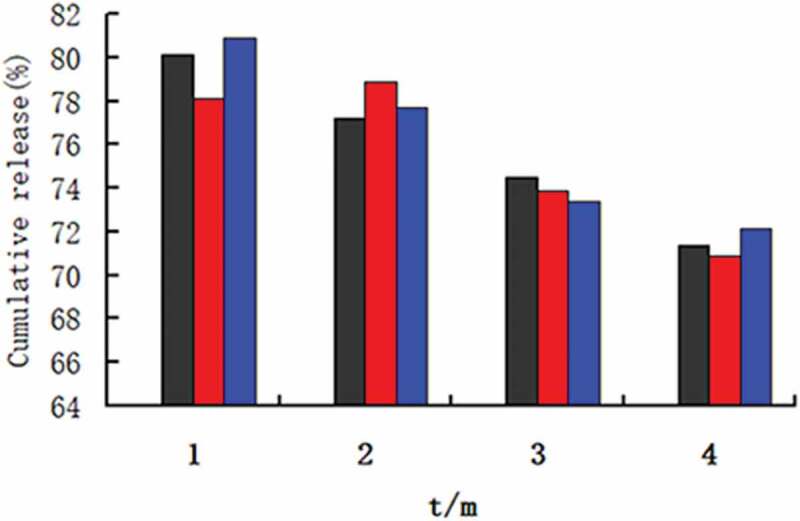
Cumulative release rate of Pue-loaded HA-ADH-PS nanomicelles at different acceleration times

Figure 14 shows that the drug release property of samples at different acceleration time. The same sample was conducted three parallel drug release experiments. It could be seen from Figure 14 that the cumulative drug release of the same sample of 72 hours showed a downward trend, but there was no significant difference. Moreover, the cumulative drug release at different acceleration time was more than 70%. The results showed that Pue-loaded HA-ADH-PS nanomicelles had good stability.

### 3.7. In vitro drug release of puerarin from pue-loaded HA-ADH-PS nanomicelles

**Figure 15.**
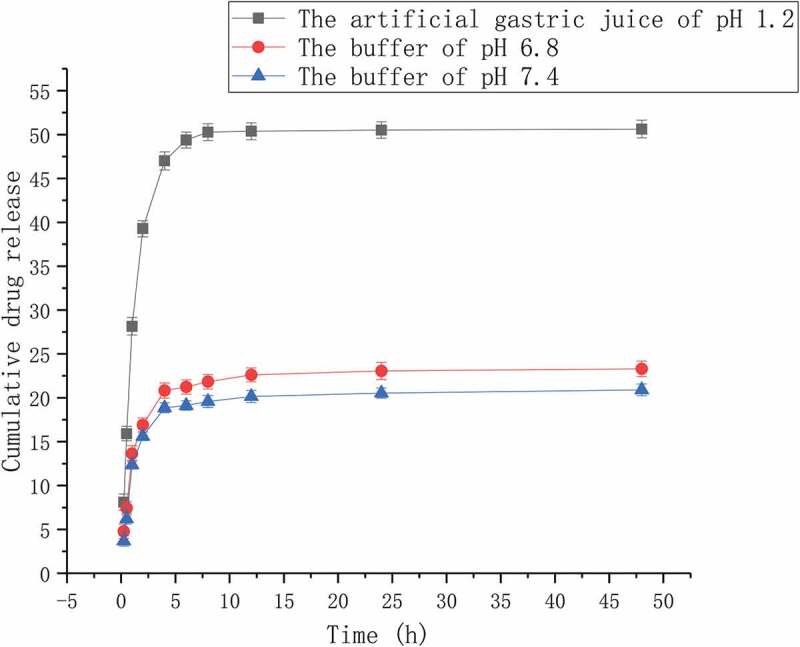
In vitro drug release profile of Pue-loaded HA-ADH-PS nanomicelles

The release behaviour of Pue-loaded nanomicelles was exploited under the buffer of pH 6.8, the buffer of pH 7.4 and the artificial gastric juice of pH 1.2. Figure 15 shows the release curve of three kinds of Pue-loaded HA-ADH-PS nanomicelles. The drug release of Pue-loaded HA-ADH-PS nanomicelles was indirectly obtained by the measurement of the samples absorption using a UV spectrophotometer at 250 nm. As illustrated in Figure 15, the vitro drug release behaviour of Pue-loaded HA-ADH-PS nanomicelles was obviously related to the pH value and the medium, and the drug release in artificial gastric juice was significantly higher than the PBS buffer of in pH 6.8 and 7.4. Within 8 hours, the cumulative drug release rate was 50.28% in artificial gastric juice, 21.83% in pH 6.8 PBS buffer, 19.57% in pH 7.4 PBS buffer; Within 48 hours, 50.62% in gastric juice, 23.30% in pH 6.8 PBS buffer and 20.90% in pH 7.4 PBS buffer. Compared with PEGylated mesoporous silica nanoparticles, the release of Pue has been improved [3]. The release of puerarin from HA-ADH-PS carriers could be divided into three stages. A burst release was in the first hour, puerarin adsorbed on HA-ADH-PS nanomicelles by non-covalent bond was released rapidly. A slow and continuous release was from 1 h to 12 h, puerarin connected on HA-ADH-PS nanomicelles by non-covalent bond and chemical coupling. The constant-rate release was after 12 hours, the puerarin was released continuously due to the degradation of HA-ADH-PS nanomicelles. In addition, the drug release of nanomicelles in artificial gastric juice was faster than the other two mediums, because hyaluronic acid and ester bond were sensitive to acidic environment.

### 3.8. The radical scavenging activity of Pue-loaded HA-ADH-PS nanomicelles

**Figure 16.**
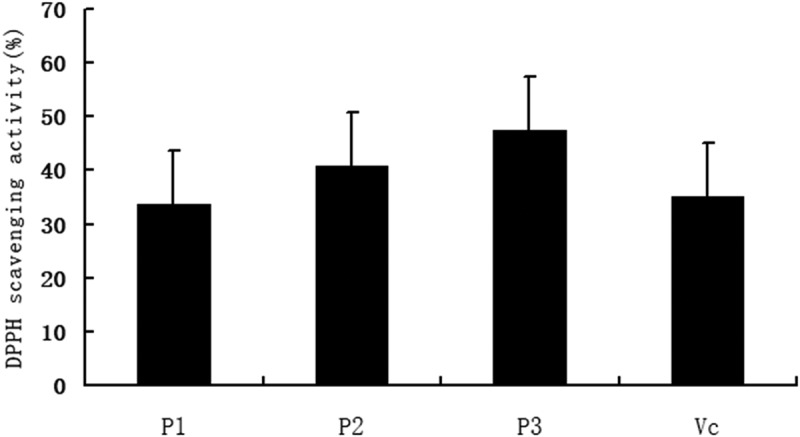
The DPPH radical scavenging activities of different concentrations of Pue-loaded HA-ADH-PS nanomicelles. All values are expressed as mean ± SD, * P < 0.05, **P < 0.01 vs Vc (n=6), P1, 15 µg/ml Pue-loaded HA-ADH-PS nanomicelles; P2, 30 µg/ml Pue-loaded HA-ADH-PS nanomicelles; P3, 60 µg/ml Pue-loaded HA-ADH-PS nanomicelles; Vc, 200 µg/ml vitamin C

The DPPH radical scavenging activities of Pue-loaded HA-ADH-PS nanomicelles were presented in Figure 16. As shown in Figure 16, the DPPH radical scavenging rates of 15 µg/ml、30 µg/ml 、60 µg/ml of Pue-loaded HA-ADH-PS nanomicelles were (33.6 ± 5.1)%, (40.7 ± 7.2)%, (47.5 ± 8.1)%, respectively. Compared with the positive control group, the Vc group (35.1 + 7.9), P < 0.05 or 0.01, which suggested that Pue-loaded HA-ADH-PS nanomicelles had an obvious scavenging effect on DPPH radical.

### 3.9. Biocompatibility study

**Figure 17.**
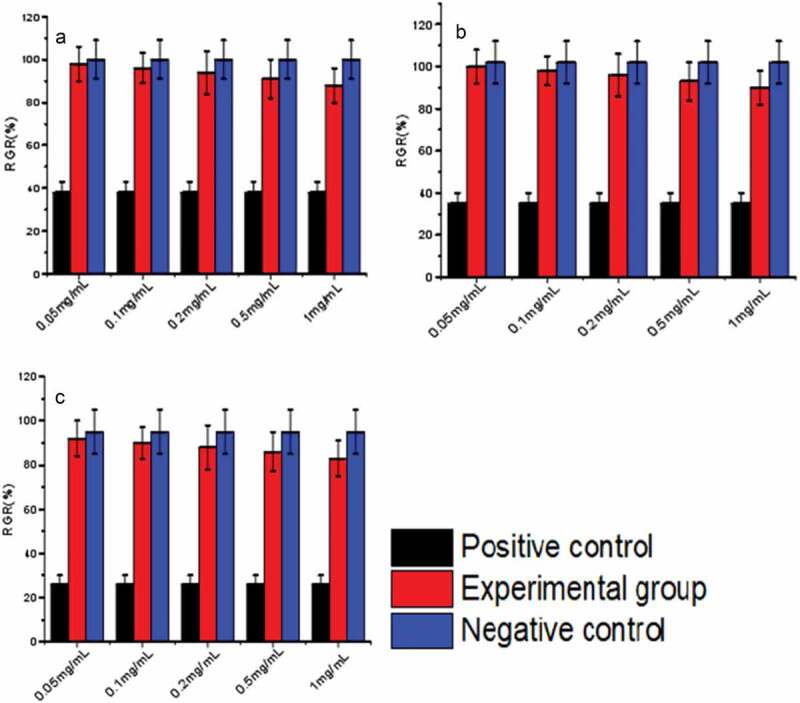
Relative activity of L-929 cells after 24 h (A), 48 h (B), and72 h (C) culture with HA-ADH-PS nanomicelles solutions at different concentrations compared to the negative control. Data are presented as the mean士SD (n = 3)

Figure 17 shows the viability of L-929 cells co-cultured with nanomicelles for 24, 48 and 72 h. As shown in Figure 17, the viability of L-929 cells varied with culture time, and the number of L-929 cells reached its maximum the next day. Moreover, the survival rate of positive control cells was very low, while the samples had little effect on the growth of L-929 cells of negative control cells. These conclusions were further validated by the cytotoxicity assessment data in Table 4. Therefore, the sample was non-toxic and had good biocompatibility.

**Table 4.** RGR values of nanomicelles to L-929 cells during 72h of incubation, where Level represents cytotoxicity level

	24 h		48 h		72 h	
group	RGR(%)	Level	RGR(%)	Level	RGR(%)	Level
0.05 mg/mL	98 ± 8	1	100 ± 8	0	92 ± 8	1
0.1 mg/mL	96 ± 7	1	98 ± 7	1	90 ± 7	1
0.2 mg/mL	94 ± 10	1	96 ± 10	1	88 ± 10	1
0.5 mg/mL	91 ± 9	1	93 ± 9	1	86 ± 9	1
1 mg/mL	88 ± 8	1	90 ± 8	1	83 ± 8	1
the negative control group	100 ± 9	0	102 ± 10	0	95 ± 10	1
the positive control group	38 ± 5	3	35 ± 5	3	26 ± 4	3

## 4. Conclusion

In this paper, HA-ADH-PS was prepared with EDC, NHS and DMAP as catalysts and adipic dihydrazide as linker. Their structures were characterized by IR, UV and TG. A series of characterization results showed that HA-ADH-PS nanomicells were a potential drug carrier for puerarin.
